# Clinical Remarks upon Surgical Cases in the Buffalo General Hospital—Excision of the Hip-joint—Removal of Fatty Tumor

**Published:** 1866-02

**Authors:** J. F. Miner


ART. III—*Clinical Remarks upon Surgical Cases in the Buffalo General Hospital — Excision of the Hip-joint —Removal of Fatty Tumor.* By J. F. Miner, M. D.

Gentlemen:—Your attention is first called to the condition of the child from whom we exsected so large a portion of the humerus. The success of this operation has been so far, complete; the child has at no time been made very sick; the discharge of pus has greatly lessened, and the parts look now as though it would soon cease entirely: the arm has shortened up considerably, but the motions of the forearm and hand are nearly perfect, and it is now giving promise of the most satisfactory results.

I will also fulfill my promise of reporting results in the case of *lumbar abscess* treated by puncture. It will be recollected that differences of opinion upon the point of treatment were announced and explained, at the time of the operation, and on that account partly, I return, to report the result so far as it could be determined by the few weeks he remained in the hospital after it was made. From the time the pus was allowed to escape, there was an entire freedom from all pain; he was soon able to assume the erect position, and no longer stood stooping. Pus continued to escape in small quantities for about ten days and then ceased entirely. The patient remained for a few weeks to assure himself that he was well, and in obedience to advice to keep quiet; he then left, declaring himself as well as ever. Remember now, that *I* do not report him well; that he feels well, is not proof that his malady will not return to torment him, and if the pus did arise, as was supposed, from disease of the vertebræ, lumbar abscess or psoas abscess will yet again appear, and our patient will be convinced that he is not well. I should be most happy to be able to report perfect recovery of so grave a malady, but I would rather warn you not to form your opinions too hastily, or too early conclude that your treatment is productive of cure. If this patient, who now appears to be quite well, has no return of this trouble, it would be natural to think, and say, that early puncture had been productive of cure, in a case of lumbar abscess, but I shall propose other explanation which, though it gratify professional pride less, is probably much nearer the truth. It is more probable that we have made mistake in diagnosis—that we have called a simple abscess by a worse name, and supposed it had its origin in caries of the vertebræ, when in reality it was not connected with such disease, rather than that any plan of treatment should have so readily cured a disease consisting in organic change. The result then, though it now appears favorable, may yet prove otherwise. If it should continue, it throws doubt upon the diagnosis, and does not prove anything, only that we punctured what we supposed was lumbar abscess. If it was, it will almost certainly re-appear; but whatever might have been its nature the operation which was made under some protests, was productive of good, was undoubtedly proper and desirable; the results show this—show that the treatment was proper, but still leave many other points in doubt.

*Excision of the Hip-joint or of the head and neck of the Femur.* Excision of the hip-joint as it has generally been called, is an operation so rarely made, and one attended by so much danger to life that I will preface by a brief history of this case, from the commencement of the disease up to the gresent time.

Mary Frederick, when about six years old, without any known injury, commenced to complain of pain in the knee-joint, and to avoid motion of the affected leg. She had previously been quite healthy, and there was no supposed constitutional bias to disease of any kind. In a few weeks after the first complaint, I was called to see and advise in the case. An extension splint was carefully applied and faithfully tested. At first it seemed productive of some relief from pain, but it was soon apparent that suppuration was taking place, and that the accumulated pus must be allowed to escape; puncture was made, and quite a quantity of offensive pus discharged. Suppuration never ceased from that time, and if the exit of pus became obstructed, pain was severe until free escape was allowed. Opium has been the chief dependence for relief of pain, and motion in the effected joint has been for the most part impossible on account of the severity of the pain it produced.

About two years from the commencement of the disease the patient is presented before you for removal of whatever of dead bone may be found. The history and appearances are so characteristic as to leave no doubt of the presence of disease of the structures entering into the composition of the joint; that the acetabulum is, or is not, involved in this disease, is impossible to determine, and I have never made much effort to ascertain the condition by probing, being perfectly satisfied by the appearances—by the position and motions of the foot and leg, and the continued discharge of pus—of the presence of disease of bone. When our patient is fully under the influence of chloroform, we shall be able perhaps to judge somewhat of the amount of disease of the joint, by the motions of the roughened and irregular articular surfaces. In my mind there is no doubt of the propriety of the operation we are about to make; its results are uncertain, still there is no ground of expectation without it. I have for several months considered the necessity as sufficiently settled and have fully determined to try the chances it offers. It is a private patient, admitted this morning for the operation, thus affording no opportunity for consultation with my colleagues, and consequently no one of them should be held responsible for advising it.

A longitudinal incision, you observe, is made about three inches in length, passing directly over the point of the great trochanter; the periostium is carefully separated from the bone, to below the diseased portion, and the head, neck and great trochanter removed. The hemorrhage is exceedingly small, and no ligatures are required. The cavity of the ascetabulum is not diseased, but you will observe that the head of the femur is nearly destroyed by the ulcerative process which has been going on. The specimen is a representative one, and whatever may be the results of the case, will be valuable and instructive.

Left to themselves such cases nearly always prove fatal, life being destroyed by the slow processes of exhaustion and hectic irritation. If, then, excision offers any ground of expectation, certainly our little patient is entitled to the benefit. In this case though the disease is extensive in the head of the bone, still it is confined to it, and does not extend to the ascetabulum, and I have great expectation of favorable results; it appears probable to me, that such disease of bone may be removed without great risk of life—that the operation will prove successful in nearly as great a proportion of cases, as other capital operations. I shall dress this leg with simple water dressing, and shall apply an extension and counter-extension splint which I have extemporized from a crutch, one side cut out so as not to press upon the thigh, and admit of ready dressing. I have a splint made for the purpose, but as the one described looks so nicely I shall use it for the present. It has advantages over all others; it is simple and convenient, it is yet efficient and satisfactory. Any dressing which will keep the leg moderately extended will answer all indications It will not be attempted to keep it extended as long as the other; it will contract, but if the disease disappear we shall believe we have saved our patient from death, and be thankful that the shortening is not much greater, rather than complain because there is any. This case will interest you all, and I will see that you have a report of the results, either verbally or through the pages of the *Buffalo Medical and Surgical Journal.*

*Fatty Tumor.*—These growths are quite common, appearing upon almost all parts of the body. This one, upon the outer aspect of the leg, has grown so large that the patient desires its removal mainly on account of its weight in walking, and the inconvenience of having it dangling from the leg. These tumors are peculiar, exactly similar to natural fat in their structure; there are but few vessels in their composition, and consequently unattended by much hemorrhage in removal. The medical treatment of such growths is wholly unsatisfactory, and it is better to attempt nothing in that direction.

A great variety of medicines have at different times been prescribed. Iodine now standing at the head of medicines of its class has been greatly used for such purposes, but as before remarked, medical treatment is useless. Surgical interference is more proper, and is often attended by satisfactory results.

